# The role of leadership in HRH development in challenging public health settings

**DOI:** 10.1186/1478-4491-6-23

**Published:** 2008-11-04

**Authors:** Judith Schiffbauer, Julie Barrett O'Brien, Barbara K Timmons, William N Kiarie

**Affiliations:** 1 Management Sciences for Health, Cambridge, MA, USA; 2 Crystal Hill Consulting, Nairobi, Kenya

## Abstract

As part of the special feature on leadership and human resources (HR), Management Sciences for Health profiles three leaders who have made a significance difference in the HR situation in their countries. By taking a comprehensive approach and working in partnership with stakeholders, these leaders demonstrate that strengthening health workforce planning, management, and training can have a positive effect on the performance of the health sector.

Three profiles are presented, from Afghanistan, South Africa, and Southern Sudan, revealing common approaches and leadership traits while demonstrating the specificity of local contexts.

## Introduction

As part of the special series on leadership and human resources (HR), Management Sciences for Health profiles three leaders who have made a significant difference in the HR situation in their countries. By taking a comprehensive approach and working in partnership with stakeholders, these leaders demonstrate that strengthening health workforce planning, management, and training can have a positive effect on the performance of the health sector.

## Discussion

### Afghan Leadership in Human Resources for Health: Overview

#### Problem

Afghanistan lacks health care providers, especially female providers – in a traditional culture in which women can be cared for only by other women – and many workers' skills need to be upgraded.

#### Approach

The Director of Human Resources (HR) at Afghanistan's Ministry of Public Health (MOPH) formed a leadership group that includes representatives from throughout the government, professional associations, unions, universities, and civil society. This group established a Directorate of Human Resources and helped link its work to that of other departments. The Directorate established a national registration system and a database of health workers. A board was established to test and certify staff in nine categories of nursing, midwifery, and allied health.

#### Local Setting

After decades of conflict, Afghanistan has some of the worst health indices in the world. The maternal mortality ratio translates into a lifetime risk that one in seven women will die of complications of pregnancy and childbirth [1]. Twenty-six percent of children will die before their fifth birthdays [2]. The country is bisected by high mountains that make transportation and access to services very difficult.

#### Relevant Changes

The HR database now contains information about 24 500 health workers, 2400 of whom have been tested for certification. Nursing and midwifery curricula have been revised, and an accreditation system for midwifery education is in place. Afghanistan's Institute of Health Sciences had graduated 800 professional midwives, the first trained in Afghanistan in seven years, by September 2006; two years later, this number exceeded 1100. The Directorate of Human Resources also established a competitive, transparent system for civil service recruitment.

#### Lessons Learned

Changes that require working across the government are complex, but working together to achieve consensus has been successful in advancing HR for health in Afghanistan. It has been important to elevate the HR function in the MOPH and establish mechanisms for interdepartmental work on HR.

#### Leadership Profile: Dr. Bashir Noormal

From 2003 to 2006, Dr. Bashir Noormal served as Director of Human Resources at the MOPH, where he and his staff laid the foundation for developing and managing HR in a country facing some of the world's greatest health challenges. The Ministry works with many partners, including the Aga Khan Foundation, the Japan International Cooperation Agency, and Management Sciences for Health.

"Afghan health statistics are heartbreaking," says Dr. Noormal. "One of the most tragic is a maternal mortality rate of over 1600 per 100 000 live births. When it comes to dealing with this and other health problems, human resources are paramount. In Afghanistan, where for two decades war interrupted – and often utterly prevented – the education of health providers, we have two particularly severe obstacles to overcome: our number of health care providers – especially females – is too small, and the quality of our providers is too low."

To address these challenges, Dr. Noormal formed a leadership group that included managers from different levels of the MOPH, representatives from the Ministries of Education and Finance and the Civil Service Commission, professional associations, unions, and universities. His team also included representatives from civil society, which delivered health services during the years of conflict.

Because of the urgency of HR issues in Afghanistan, one of the first things the group proposed was elevating the HR function to the directorate level in the Ministry. This change would enable it to coordinate all aspects of HR, which were previously spread throughout the MOPH. Once the Directorate of Human Resources was established, it defined how this unit would work with the other units that had previously covered HR functions, for example, NGOs that are contracted to deliver primary health care services through the Grants and Contracts Management Unit of the MOPH. This resulted in what Dr. Noormal calls the "linkages model." This model was useful in showing what combination of ministry departments could be drawn together to address common goals and how to best link to the provinces.

With an organizational basis for HR established, one of the Directorate's first tasks was to identify the numbers, types, and locations of health workers in the country and determine their level of competency. Among the many refugees returning to Afghanistan were health workers trained by NGOs operating cross-border projects in Pakistan during the war, who, out of necessity, had expanded their roles beyond their training. Others had no formal training but nevertheless provided health care during the war years. Still others may have received training irrelevant to their current jobs.

To address this array of health workers and begin to build a consistent level of quality into the HR system, the Directorate of Human Resources put in place a national registration system and created a database that details the training and background of 24 500 health workers. A semi-autonomous board was established to test and certify staff in nine categories of nursing, midwifery, and allied health. Approximately 2400 people have been tested to date.

While it is imperative to increase the number of Afghanistan's health workers, the quality of their training is also crucial. Dr. Noormal cites an Afghan proverb to make this point: "One excellent soldier is better than an entire army with poor skills."

High-quality training is being provided to refresh the skills of health workers and train new ones, especially female doctors, nurses, and midwives. Both nursing and midwifery preservice curricula have been revised. An accreditation system for nursing education is being finalized, and a system of accreditation for midwifery education has been established. With these new standards, Afghanistan's Institute of Health Sciences had graduated 800 professional midwives, the first trained in Afghanistan in seven years, as of September 2006. By September 2008, this number had reached 1128.

Under Dr. Noormal's leadership, the Directorate of Human Resources also established a competitive, transparent system for civil service recruitment. As the only ministry to have established these systems and standards, the MOPH has been identified by the Afghanistan Civil Service Commission as one of the lead ministries in the government to implement civil service reform.

Dr. Noormal knows that the measures the Directorate has taken are not the sole solutions to Afghanistan's health problems. Once a cadre of well-trained health care providers exists, men and women must still be recruited and deployed to rural areas, where they are desperately needed.

With the help of donors, the MOPH has instituted a system of generous salary incentives to induce health care providers, especially women, to work in rural areas, says Dr. Noormal, "but," he adds, "such incentives will remain insufficient unless other measures are also taken." Security must be strengthened in all areas of the country. In addition, female health care providers will traditionally not relocate without their families, which means there must be schools for their children, employment for spouses, and adequate housing. "These things," Dr. Noormal points out, "are not under MOPH control, but rather require the cooperative efforts of the entire Afghan government."

Dr. Noormal's approach to achieving the gains made by the Directorate of Human Resources has been characterized by a process of reaching consensus and developing and instilling a shared vision. Ideas and issues discussed and agreed upon by the HR Taskforce are taken to senior MOPH officials. Matters of policy and guidelines are shared with stakeholders before they are approved by the MOPH Executive Board. Implementation has taken place with the help of donors and MOPH partners. "Their support has been a vital component of our successes," states Dr. Noormal.

Still exhibiting the energy and determination that have sustained him throughout these formidable tasks, in June 2006, Dr. Noormal turned over the reins to Dr. Salam Jalali, former director of Kabul's Indira Gandhi Institute. The two men fully agree on the importance of continuing and building upon the HR systems that the MOPH established during Dr. Noormal's tenure.

Having reassumed his position as Director of Human Resources Development for the World Health Organization's office in Afghanistan, Dr. Noormal continues to support improvement in the number and quality of Afghanistan's health workers. As the WHO representative on the Ministry's HR Taskforce, he remains actively involved in developing and improving HR management at the MOPH. Dr. Jalali, who values his input, invited him to serve as an advisor to the Directorate of Human Resources.

"The human resource work we have begun is of the utmost importance," states Dr. Noormal. "Our priority is to improve the health of all Afghans, and to do so, Afghanistan must develop its human resources to the fullest and manage them wisely."

### Emergency Health Workforce Planning in South Africa: Overview

#### Problem

South Africa's health system is facing a shortage of trained health staff of serious proportions. In rural communities where the spread of HIV & AIDS is escalating, providing an adequate pool of qualified health professionals is difficult. Low salaries and poor working conditions deter people from pursuing public health professions, exacerbating the problem of migration of health workers to the private sector and wealthier countries.

#### Approach

Under the auspices of South Africa's National Department of Health (NDOH), Dr. Percy Mahlathi, Deputy Director General of Human Resources, led a multisectoral team to identify the sources of South Africa's human resource (HR) challenges. Using the World Health Organization's HR Toolkit (2004) as a basis for developing a strategic framework, the project team developed a National Human Resources for Health Plan, which provides national guidelines for HR development, management, and training [3].

#### Local Setting

According to the South Africa Institute for Race Relations, the public sector had only 7645 doctors (of the 30 000 registered). The Institute found the number of doctors "alarmingly low": "the local doctor to population ratio was 0.7 doctors per 1,000 people – as opposed to 2.1 in Egypt and 1.2 in the Philippines" between 1994 and 2004 [4]. While South Africa is committed to recruiting and training highly qualified health care managers, it continues to grapple with the migration of these people to more developed nations. Since the end of apartheid, career opportunities outside health have increased, as has competition for top students. Many look outside health care – a field that historically was the only career path for black South Africans – because they have witnessed the hard work and poor conditions that their parents endured for very low wages. Both the Departments of Health and Education are looking for ways to change this image and promote health sciences as a career option.

#### Relevant Changes

According to the Department of Health's 2007 annual report, significant progress has been made in HR in four provincial Departments of Health: a new remuneration system for health professionals, policies and curricula for mid-level health workers, training for Clinical Associates, and strategies to strengthen the nursing profession are in place [5]. Nationally, a community service program has decreased migration of new graduates, 3800 of whom have been deployed to rural areas.

#### Lessons Learned

Although progress has been made, many challenges remain and addressing them requires a multisectoral approach. Strong health management and leadership and additional human and financial resources will help meet the needs of South Africa's citizens.

#### Leadership Profile: Percy Mahlathi

More than 13 years after gaining independence and holding its first democratic elections, South Africa has much to be proud of. The reconstruction, nation-building, and democratization that ensued during the past decade enabled the merging of various states and authorities into one unified "rainbow nation." With gains in some development indicators and a growing economy, South Africa has become an influential country, not only in the region but increasingly throughout the world.

Despite these achievements, South Africa has one of the highest numbers of people living with HIV in the world, with a 2008 estimate of 5.7 million or almost one in five adults [6]. While the country is no longer separated along racial lines, South Africa's health system retains many inequities from the apartheid era, and a major challenge for the government is to improve the accessibility and quality of basic health services for its majority population.

Dr. Percy Mahlathi of South Africa was the only one in his elementary school class to attend high school. That he made it through medical school during a time when South Africa struggled with extreme social inequities is a testament to his leadership, commitment, sense of justice, and drive. That he is currently preparing leaders to build South Africa's health sector while making significant contributions to world health is admirable.

Dr. Mahlathi, Deputy Director General of Human Resources for South Africa's national Department of Health (DOH), took office in 2004 – exactly 10 years post-apartheid. In this role, he is charged with counteracting the ever-present brain drain; ensuring that health workers are experienced and competitively paid; improving working conditions; and seeing that rural communities have access to a consistent supply of well-trained health professionals who can provide primary health services while tackling the AIDS epidemic.

To address the migration of health professionals, Dr. Mahlathi reached out to others both within and outside the national DOH. Together they scanned the environment and analyzed the root causes of their most critical challenges. This analysis enabled Dr. Mahlathi to finalize a national plan for HR management. But he realized that for the plan to be successfully implemented, the process needed to be guided by skilled leaders and managers at all levels.

#### Creating a Successful National Plan

As part of the national efforts, Mahlathi distinguishes five key points as essential to guide the implementation of the national HR plan for health:

HR development: Develop highly qualified health leaders and professionals, including HR managers.

Multisectoral planning: Create coordinated plans with the private and public sectors, encompassing education, economic development, justice, transportation, and communication.

Harmonization in the public sector: Work with the National Treasury and Department of Education to lead the provinces in addressing their issues through a 'harmonized' approach.

Working with trade unions: Because South Africa's health services depend on people, prioritize quality of care and health workers' competence while working with trade unions to improve working conditions and remuneration for health workers.

International leadership: Since health systems throughout the world are interdependent – as avian influenza, AIDS, and tuberculosis have shown – South Africa must take a leadership role and produce more opinion leaders.

#### Results of the Plan for HR for Health

Many health workers continue to look outside Africa for career opportunities. Highly skilled health professionals are migrating to Saudi Arabia, New Zealand, the United Kingdom, and Canada in record numbers [7]. Compelling them to stay poses an ethical question that challenges South Africa's democratic constitution, which recognizes its citizens' rights to determine their future and move anywhere in the world.

One way in which the DOH has addressed the rapid migration of new graduates, short-term need for health care workers, and the lack of health workers in rural areas has been introducing a program that requires health students to perform community service before they can be registered by the Medical and Dental Board of South Africa or South African Nursing Council [8]. In the beginning, this policy was unpopular. Many who grew up in urban settings did not want to travel and live in rural communities where there was little to do. Once there, however, many health professionals, from pharmacists to dentists, found the work fulfilling. Mahlathi spoke proudly about two young medical school graduates from Johannesburg who were assigned to Limpopo Province. One year later they asked for an extension, stating that the "people were so generous and respectful."

Today, there are more than 3800 health professionals working in the community service program. The success of the program has impressed others in the public sector: both the justice and engineering sectors are looking to community service as a means of addressing their short-term staffing issues.

In a country where the burden of HIV & AIDS is intensifying, Mahlathi knows that realizing his vision requires developing strong HR leaders. "We cannot aspire for mediocrity," Mahlathi asserts. "We need to leverage the skills of the people we have by developing strong leaders in programs that have been cooked at home – leaders who can clearly articulate and execute South Africa's health vision and planned outcomes." He believes that programs initiated and led internally are more relevant than foreign ones because they build confidence in South Africa's vision and strengthen the country's capacity in HR management. Mahlathi is also confident that South Africa today has the ability and the resources to achieve its goals. But all actions must be directed toward building strong HR management systems and increasing public awareness and the appeal of the health care industry.

"South Africa has always had good opportunities," he points out. "We have always had good resources and skills. But we've never had a situation where we have effectively managed the human resource crisis in health. We must provide our future health leaders with opportunities. Then South Africa will succeed."

### Leading the Development of Human Resources for Health in Southern Sudan: Overview

#### Problem

Sudan needs to scale up health services despite serious shortages of workers in all health professions: there are only slightly more than 100 doctors and 5 pharmacists to serve more than 10 million people scattered throughout a huge country with virtually no road network [9].

#### Approach

The Director for Human Resource Development and Planning in the Ministry of Health (MOH) of Southern Sudan is leading the achievement of public health goals by collaborating with stakeholders, focusing on HR priorities, mobilizing resources, and building leadership and management capacity.

#### Local Setting

Sudan has some of the worst health indicators in the world. Almost 1 in 10 Sudanese children die before reaching their fifth birthdays, while maternal mortality is estimated at 590 per 100 000 live births. In Southern Sudan, malaria, acute respiratory tract infections, and diarrhoeal disease are major killers. Only 34% of eligible children are immunized against measles, and 94% of deliveries take place at home [10].

#### Relevant Changes

After an HR assessment, Southern Sudan's MOH made progress in addressing the shortage of workers by training health managers in HR management and leadership; repatriating doctors from Canada and recruiting health workers from East Africa; scaling up training of mid-level workers; developing an HR policy; developing preservice training curricula; and establishing a national HR information system.

#### Lessons Learned

Education of health workers and professionalization of management and leadership are crucial. Coordinating the activities of donors and partners is equally important to produce health workers to meet the needs of the country.

#### Leadership Profile: Dr. Monywiir Arop Kuol

In Southern Sudan, as in many countries that are grappling with a critical shortage of health workers, dynamic HR directors make a difference. Dr. Monywiir Arop Kuol is such a leader. He is contributing to achieving public health goals by working collaboratively with stakeholders, focusing on HR priorities, mobilizing resources, and enabling others to lead and manage in order to achieve sustainable results.

Although drought, famine, poverty, and war have afflicted Sudan for decades, Sudanese leaders in public health have a vision of a better future. A general practitioner, Dr. Monywiir knows these problems well, since he has been managing relief services in Sudan since 1990. Dr. Monywiir currently serves as Director for Human Resource Development and Planning in the MOH of Southern Sudan. In this role, he is responsible for policy development, planning, coordination with many partners, supervision, and monitoring and evaluation.

In his 20-year career in health systems and services, Dr. Monywiir has done everything from community mobilization to fundraising. During the civil war, he spent many years in the bush. His responsibilities ranged from treating wounded soldiers to managing education programs in the areas controlled by the Sudan People's Liberation Army. Dr. Monywiir is passionate about helping young people – he would like to see the many brilliant and hard-working young people, especially those who served with him and missed opportunities to go to high school and college, be able to acquire useful skills and education.

In high school, Dr. Monywiir dreamed of becoming an engineer. When the time came for him to go to the university, however, the community elders unanimously decided that he should study medicine, so young Monywiir headed off to the University of Juba in Southern Sudan. Although he was not consulted, Dr. Monywiir confesses that he grew to like the subject and graduated at the top of his class.

More recently, Dr. Monywiir has established and operated relief agencies, coordinated the activities of health NGOs, and worked for the International Rescue Committee in Sudan. His vision is for Southern Sudan to have "efficient, equitable, and advanced health services provided by a highly skilled and motivated workforce."

Since the peace agreement was signed between the national government of Sudan in Khartoum and the Sudan People's Liberation Movement in the south in 2005, donors have committed US$ 484 million to the trust funds of the two governments. In the south, these funds are being used to expand basic health services for the 75% of the people of Southern Sudan who lack access to services. Donors are helping to develop the capacity of the MOH and are investing in infrastructure, pharmaceuticals, and equipment [11].

Against this backdrop, Dr. Monywiir recognized the need to collaborate with all relevant partners – including the World Bank, World Health Organization, United Nations agencies, NGOs, civil society, the private sector, and the ministries of education and public service – to produce health workers. He established a leadership team that included members of his staff and representatives of donor agencies and technical assistance organizations. Among Dr. Monywiir's goals for this team was to harmonize the activities being proposed.

As part of its initial scanning, the team surveyed existing health services and staff and collected data about the elements of an effective HR management system. Including HR management issues in the baseline survey proved invaluable as Dr. Monywiir and his staff began to organize HR functions and the policies to support them.

The baseline assessment identified many HR problems – among them, low salaries, unavailability of jobs, lack of infrastructure, and low levels of education – and enabled the leadership group to identify priorities. As Sudan emerges from two decades of civil strife, it needs to attract health workers back into the country, which has only about one health worker per 1000 people [12]. Dr. Monywiir is leading efforts to attract health workers to return. Sudanese doctors in Canada have come back to Southern Sudan to work in public and NGO health facilities.

Some of these problems cannot be addressed directly by the MOH, but in keeping with Dr. Monywiir's collaborative approach, a mechanism is already in place to support action on these critical fronts. Meanwhile, he and his team have focused on:

▪ creating a plan for human resources for health, with an emphasis on training midwives (who are urgently needed to help lower the maternal mortality rate);

▪ developing an HR policy;

▪ addressing gender inequalities;

▪ developing HR and leadership capacity among managers at the central and regional levels.

The last priority exemplifies Dr. Monywiir's visionary approach. He recognizes that without professional HR leaders and managers throughout the system, his dreams for improving health in his country will fail. With the support of USAID and other partners, the MOH's HR directorate has been involved in developing the HR management and leadership skills of public- and private-sector health managers. In 2006, a workshop for stakeholders in human resources for health was held in Juba, Southern Sudan. Participants were introduced to best practices in HR management and new models for leading and managing: the Global Health Workforce Alliance's Human Resources for Health Framework and MSH's Leading and Managing for Results Model. A program is underway to train health managers from various regions in Southern Sudan in HR and leadership skills. To build his own knowledge, Dr Monywiir attended a one-month course on human resources for health at the University of New South Wales in Australia.

Dr. Monywiir asserts that in the new Southern Sudan, "Human resource management policies and practices will have to be innovative, humane, and competence based." He and others in the MOH are supporting this evolution by developing HR policies, plans, and guidelines. A national HR policy has been finalized, and work on a 10-year strategic plan has begun.

Other initiatives include establishing and rehabilitating training programs and institutions in Sudan (which has 14 medical training institutions) and neighboring countries. Nurses, laboratory technicians, and community health workers are being trained to upgrade their skills and enable them to train new health workers. Southern Sudan is also recruiting health workers from "the rich regional workforce market of East Africa, where there is immense expertise and a culture of hard work," explains Dr. Monywiir. To provide accurate and timely data on the progress being made, the MOH has developed a comprehensive HR information system, which will greatly improve HR planning and management.

Dr. Monywiir is committed to help rebuild the country and use its abundant natural resources by making a healthy workforce available so that social-sector services can be re-established. To date, he and his team can point to the following achievements:

▪ more than 30 health managers trained in HR management

▪ more than 30 health managers trained in management and leadership

▪ HR policy developed

▪ training of mid-level health cadres scaled up

▪ preservice training curricula for health workers developed or standardized

▪ nationwide HR information system developed

▪ 15 doctors repatriated from Canada.

## Conclusion

HR leaders show the way through their commitment and creativity, informed by experience about what works. In Afghanistan, South Africa, and Southern Sudan, leaders have succeeded in very different settings by using some common strategies: a multisectoral approach and comprehensive planning; development of an HR policy; establishment of a dedicated HR unit and training of HR managers; expanded recruitment and training, testing, and certification of health workers; revision of preservice training curricula; and a nationwide HR information system.

## Competing interests

The authors declare that they have no competing interests.

## Authors' contributions

JS wrote the section of this article on Afghanistan, while JBO wrote the section on South Africa, and WNK drafted the section on Southern Sudan, for which BKT provided research and writing. BKT edited the entire article. All authors read and approved the final manuscript.
